# The acquisition of expertise in the classroom: are current models of education appropriate?

**DOI:** 10.3389/fpsyg.2014.00580

**Published:** 2014-06-12

**Authors:** Craig P. Speelman

**Affiliations:** School of Psychology and Social Science, Cognition Research Group, Edith Cowan UniversityJoondalup, Australia

**Keywords:** expertise, skill, numeracy, mathematics, computer games

## Introduction

The study of expertise tends to focus on humans who can perform extraordinary feats. Although the way in which expertise is acquired is often characterized as similar to everyday skill acquisition, the attainment of basic numeracy skills is rarely considered in the same context as the attainment of expertise. It is clear, though, that average numeracy skills possess all the hallmarks of expert performance. In this paper I argue that the traditional classroom of Western education systems pays insufficient attention to the idea that effective numeracy skills represent a level of expertise that requires a particular form of training. Using the five principles of skill acquisition identified by Speelman and Kirsner (2005), I argue that the modern classroom is not the most appropriate environment for acquiring important cognitive skills, and that computer programs, such as games and tailored training tasks, should be considered a valuable adjunct to traditional didactic instruction.

## The nature of expertise

Experts in most fields are characterized as people who have more knowledge and abilities than non-experts. In cognitive science, this superiority in knowledge and abilities has been reported as better memory, more extensive knowledge, and highly developed procedural and perceptual skills. More often than not, experts are described as people who can perform extraordinary feats of memory and cognition, whilst possessing the same cognitive apparatus as non-experts. Their superior performance is typically explained as the end product of skill acquisition through many years of dedicated practice in their field.

## Acquisition of expertise

Based on over 100 years of research on skill acquisition and expertise (e.g., Anderson, 1982; Logan, 1988; Ericsson et al., 1993), Speelman and Kirsner (2005) identified five principles of skill acquisition that explain how expertise is associated with superior feats of cognition. In brief, the five principles of skill acquisition state that (1) practice leads to faster and (2) more efficient uses of knowledge, which enables faster performance and (3) results in less demand on mental resources. As a result, the performance of low level tasks becomes second nature, and (4) this frees up mental resources that can be utilized to attempt higher level behaviors. Ultimately (5) skilled performance reflects the development of many component processes.

## Acquisition of numeracy skills

The five principles of skill acquisition were derived from research in a broad range of fields in which people have been reported to have acquired expertise. As a result, Speelman and Kirsner (2005) claim that these principles apply to any area of cognition in which practice can lead to improved performance. Importantly, these principles can also explain performance improvements along the trajectory of expertise attainment, even at levels below which someone would be considered an expert. So, whereas in a discussion of mathematics experts Butterworth (2006) considered only those 1 in several million people with extreme abilities, the principles suggest we all show elements of expertise as we learn to count and add. I have been surprised to learn, however, how little of this view of skill acquisition is reflected in current education practice. In particular, the attainment of basic numeracy skills is rarely considered as a form of expertise acquisition, and nor are difficulties with the learning of numeracy skills seen as problems in the acquisition of expertise (e.g., a lack of practice). Instead, learning difficulties are often seen as resulting from some neurological or developmental disorder that adversely affects a child's ability to learn mathematics (Clark et al., 2014; Haase et al., 2014), or some systemic issue related to the school system (Ramsden, 1984; Biggs, 1999; van Kraayenoord and Elkins, 2004) or the child's culture (Whitburn, 1996) or SES (OECD, 2013). And yet, numeracy skills, particularly those related to early learning of arithmetic and number facts, share features with many examples of expertise. For example, they require many years of practice to attain, performance relies on pattern recognition of a large number of items (i.e., numbers and symbols) and retrieval from memory of a large number of facts, and expertise attainment is characterized as a transition through a hierarchy of skills (e.g., counting to addition) that can only occur when performance at the lower level has attained a particular level of skill (Pellegrino and Goldman, 1987; Neumann et al., 2013). These are all features that have been identified in experts in a range of other domains (e.g., Ericsson, 1996). It is rare to see the development of basic numeracy skills characterized in this manner in education research (e.g., Griffin, 2009; Lei et al., 2009; Yelland and Kildery, 2010; Neumann et al., 2013), and certainly my conversations with primary school teachers reveals they are unaware of such concepts.

## The traditional western classroom

The traditional model of the western classroom, with a teacher at the front delivering instruction to a class of 20–30 children, does not sit easily with the view of skill acquisition reflected in the five principles. Although a teacher may be able to effectively convey declarative and procedural information relevant to an arithmetic task (e.g., demonstrate how 2 × 4 is equivalent to 4 + 4), for a child to convert this sort of knowledge into a form of expertise requires a great deal of practice. Traditionally a child would get this practice by attempting to solve many problems like 2 × 4 = ? until the teacher is satisfied that most children have grasped the concept. But “grasping” the concept may not be sufficient. According to the five principles, what counts as “sufficient” practice is determined by what comes next in the trajectory of skill acquisition. For instance, if the “2×” problems are introduced as an extension of addition facts, it is important that the child is sufficiently skilled at retrieving relevant addition facts. If they stumble over the idea of 4 + 4 (i.e., they do not retrieve the answer quickly) while the teacher is explaining the “2×” concept, they may be forced to generate the answer by a counting method (e.g., start from 4 and count a further 4 places) (Pellegrino and Goldman, 1987). Such a counting strategy will tax working memory and as a result the child may not have sufficient working memory capacity to follow the explanation of the “2×” concept. This strategy will also take extra time and so the student may fall behind the teacher's explanation. It is difficult for a teacher to ensure that the pace of a lesson matches the learning rates of all children in the class, and also to monitor that all children have mastered a concept prior to introducing the next concept. Children who are fast learners may become bored and disruptive if the lesson is paced too slow. Children who are slower learners may be left behind by a lesson that is paced to match the learning rate of the faster learners. It would not take too many experiences of being left behind for someone to believe they just do not have a “maths brain” (Swan, 2004), or even develop a “maths phobia” (Furner and Berman, 2003). Even if the attainment of proficiency is assumed to occur through homework drills, it is likely that only accuracy is checked by a teacher, when speed of access to number facts is also necessary to avoid the problem just described.

## The decline in numeracy skills

The suggestion that the traditional classroom may lead to some children struggling with the acquisition of basic numeracy skills goes some way to explain the slide in numeracy skills in many western countries like Australia [as indicated in the PISA results of 2009 (OECD, 2013)]. The problem in Australia appears to be longstanding as 53% of the Australian adult population is functionally innumerate (ABS, 2006), which indicates that many would not comprehend a bank statement. Failures in elementary mathematics courses are likely to be compounded by a lack of confidence regarding higher level mathematics, and so many people neglect maths at the higher levels, leading to universities having to provide remedial classes for their commencing students (Healy et al., 2010; Slattery, 2010; Arlington, 2012; Maslen, 2012).

## An alternative education model

According to the five principles of skill acquisition, overcoming the problems identified here with the traditional Western classroom would require children to be presented with a structured learning program that involved instruction regarding each level of a hierarchy of concepts, interspersed with practice opportunities. Further, each child would only be introduced to the next level of a concept when they have reached some degree of fluency with the previous level. Until that point they would continue practicing with problems at the previous level, possibly with some form of intervention by a tutor to ensure their understanding of the concept is appropriate. This is probably the aim of most teachers, however the level of monitoring required to ensure each child has reached the requisite level of fluency is possibly beyond the capacity of a teacher responsible for 20–30 children in the one classroom. An alternative model would be to develop computer software in the form of games and tailored training tasks. Such software can be developed to not only provide hours of practice opportunities, but it can do so in an exciting and enjoyable manner that will hold the attention of children and provide them with the motivation to spend many hours mastering a concept (Rosas et al., 2003). Further, the software can be designed to deliver feedback on every response, and monitor the level of performance (i.e., both accuracy and response time) such that a child will be allowed to move to the next higher level of the concept when they have mastered the previous level, as is the case with computer games designed purely for entertainment (Towne et al., 2014). A recent study (Main and O'Rourke, 2011) demonstrated the benefits of such software, where the speed and accuracy of performance on a standard arithmetic test was improved for children who had played a maths game (*Dr Kawashima's Brain Training*) on a hand held games console compared to children who received standard classroom lessons.

Another computer game, *Numbeat*, has been designed explicitly according to the five principles. This game was developed to facilitate the acquisition of basic arithmetic skills in primary school children. *Numbeat* requires a player to destroy some “bad” characters on the screen, before they convert “good” characters into “bad” characters, by filling up some destruction device (e.g., a cannon) with an amount of ammunition that matches the number of “bad” characters. To achieve this aim requires the player to perform several types of mental arithmetic operations. The game is structured so that performance speed is important (e.g., levels have time limits). If a player beats the time limit for a particular level, they are considered to be sufficiently fluent with the arithmetic operations tested in that level, and so are allowed to progress to the next level of the game, which typically represents a slightly more advanced level of arithmetic. A level is repeated if the player does not meet the required performance standard. As such, the game approximates the deliberate practice of challenging tasks that is required to acquire expertise in a domain (Towne et al., 2014). In preliminary trials involving 248 children, playing this game for 10–15 min per day for 8–10 days resulted in an average 5% reduction in the time to solve simple arithmetic problems presented in a traditional manner (e.g., 3 × 4 = ?) (Speelman, 2013).

Although I do not claim that games such as *Dr Kawashima's Brain Training* and *Numbeat* will be the solution to all of the mathematical ills facing many countries, evidence that playing such games can facilitate the acquisition of arithmetic skills in primary school children is a positive step toward addressing learning difficulties in mathematics. This type of research may pave the way toward a situation where teachers can provide the necessary content lessons, and computers can facilitate the necessary practice that will enable students to master each level of a skill before tackling the next step in the skill hierarchy. Ultimately such evidence supports the argument that education in basic numeracy skills should reflect principles of expertise attainment.

### Conflict of interest statement

The author declares that the research was conducted in the absence of any commercial or financial relationships that could be construed as a potential conflict of interest.
